# Exploiting Radiation Induction of Antigens in Cancer: Targeted Drug Delivery

**DOI:** 10.3390/ijms23063041

**Published:** 2022-03-11

**Authors:** Vaishali Kapoor, Abhay K. Singh, Calvin D. Lewis, Sapna Deore, Dennis E. Hallahan

**Affiliations:** 1 Department of Radiation Oncology, Washington University School of Medicine in St. Louis, St. Louis, MO 63108, USA; vkapoor@wustl.edu (V.K.); abhaysingh@wustl.edu (A.K.S.); calvinlewis@wustl.edu (C.D.L.); 2 Siteman Cancer Center, Washington University in St. Louis, St. Louis, MO 63108, USA; 3 Department of Radiation Oncology, University of Iowa, Iowa City, IA 52242, USA; 4 Medical Guidance Systems LLC, St. Louis, MO 63110, USA; sapnad@medgyde.com

**Keywords:** antibody–drug conjugate, radiation therapy, therapeutic target, radiation-inducible

## Abstract

Therapeutic antibodies used to treat cancer are effective in patients with advanced-stage disease. For example, antibodies that activate T-lymphocytes improve survival in many cancer subtypes. In addition, antibody–drug conjugates effectively target cytotoxic agents that are specific to cancer. This review discusses radiation-inducible antigens, which are stress-regulated proteins that are over-expressed in cancer. These inducible cell surface proteins become accessible to antibody binding during the cellular response to genotoxic stress. The lead antigens are induced in all histologic subtypes and nearly all advanced-stage cancers, but show little to no expression in normal tissues. Inducible antigens are exploited by using therapeutic antibodies that bind specifically to these stress-regulated proteins. Antibodies that bind to the inducible antigens GRP78 and TIP1 enhance the efficacy of radiotherapy in preclinical cancer models. The conjugation of cytotoxic drugs to the antibodies further improves cancer response. This review focuses on the use of radiotherapy to control the cancer-specific binding of therapeutic antibodies and antibody–drug conjugates.

## 1. Introduction

Examples of the therapeutic uses of antibodies to treat cancer include unconjugated (naked) therapeutic antibodies that block the function of the target proteins such as EGFR and PD-L1 on the surface of cancer cells [1,2]. Secondly, antibody–drug conjugates consist of chemotherapeutic agents covalently linked to antibodies. The delivery of radiation sensitizers specific to cancer using antibodies to radiation-inducible antigens has recently been described [3]. The cytotoxic agents are released following endocytosis of the antibody–drug conjugate into cancer cells [3,4,5]. Alternatively, radioimmunoconjugates have great potential to achieve cytotoxicity when alpha emitters are conjugated to the antibodies. Finally, bispecific and fusion antibodies are increasingly used in the treatment of cancer [6]. Thus, one antibody can lead to the development of several different types of cancer drugs.

One challenge in the development of therapeutic antibodies is the paucity of cancer-specific antigens. For example, cancer neoantigens contain mutations that are specific to cancer (e.g., mutations in EGFR) [7]. Another example is over-expressed cancer antigens such as her2/neu or CD20 [7]. Each of these examples of cancer antigens is limited to particular cancer subtypes. Moreover, only a fraction of patients have cancer that expresses these antigens; for example, 30% of breast cancer patients show her2/neu expression [8]. Furthermore, intra-tumoral heterogeneity can be a limitation, wherein the antigen is not present on every cell within a tumor, or the antigen might be inaccessible to antibody binding. Overcoming these limitations of cancer antigens can improve the therapeutic antibody treatment of cancer.

## 2. Radiation-Inducible Antigens

Ionizing radiation is capable of causing morphological and functional alterations in the tissues [9]. Radiation oncology can play a role in developing cancer-targeted therapeutics because of the exaggerated biological response of cancer cells to oxidative stress [10]. Simply stated, cancer is substantially more efficient at responding to oxidative stress following ionizing radiation exposure compared with normal tissues. Cancer cells are more likely to survive and propagate in a stressful tumor microenvironment when “stress regulated proteins” are highly over-expressed. The first radiation-inducible proteins identified include cell adhesion molecules [11,12]. These inducible proteins are also not well suited for antibody development because their expression is limited to microvascular endothelial cells but not on cancer cells, and some are shed from cancer cells.

In contrast, the cancer cell response to radiation involving the ER stress response (ERSR) is exaggerated in cancer [13]. One of the physiological responses of cancer cells to radiation is the surface expression of some of these ERSR proteins. For example, GRP78 is highly over-expressed in cancer cells and participates in the cancer response to ionizing radiation [14,15,16]. We have found that these radiation-inducible antigens are molecular targets for therapeutic antibody development [15]. This strategy of targeting stress-regulated proteins substantially increases the number of cancer antigens that can be targeted with therapeutic antibodies. Since these cancer antigens are unregulated during standard-of-care radiation therapy, this strategy will be useful for all cancers treated with radiation. With advanced radiation technologies, cancer-specific radiation therapy allows marked elevation of cell surface antigens in cancer compared to healthy tissues.

Discovery platforms to identify radiation-inducible cancer antigens include mass spectrometry and the analysis of the differential expression of cancer proteins. Secondly, gene expression profiling can be used to identify radiation-inducible genes. The greatest limitation of these discovery platforms is that they identify all proteins and genes, and not only proteins on the cancer cell surface. An approach with fewer limitations is the use of molecular “bait” in a way that is analogous to fishing bait [17,18,19]. Peptide libraries were used to discover antigens on the cancer cell surface. This method is referred to as “biopanning” and involves the use of bacteriophage-displayed peptide libraries. This strategy has allowed the discovery of dozens of radiation-inducible antigens on the surface of cancer cells [17,20].

Once radiation-induced cancer cell surface antigens are discovered, they are next prioritized (i.e., ranked) for antibody development. The ranking of antigens is based upon the following criteria:

1. Cancer-specific;
2. Accessible to antibody binding;
3. Not shed/remained tethered to cancer;
4. Over-expressed in many cancer subtypes;
5. Endocytosis of the antibody/antigen complex is not an essential criterion for prioritization of inducible antigens, but it is ideal for antibody–drug conjugates.

Radiation-inducible cell surface proteins include glucose-regulated protein 78 (GRP78), tax-interacting protein-1 (TIP1), TATA-Box Binding Protein Associated Factor 15 (TAF15), P-selectin, ICAM1, E-selectin, and Integrin αvβ3. GRP78 and TIP1 are the lead inducible targets for drug development because they fulfill all criteria. Cell surface expression of antigens can be induced by various mechanisms. P-selectin translocated to the surface by the microtubule-dependent exocytosis of intracellular reservoirs within the vascular endothelium [21]. Ionizing radiation induces the expression of cell adhesion molecules and integrins through posttranslational modification and transcriptional induction [10,22,23,24]. ERSR plays an important role in the surface translocation of GRP78 [14,15,16].

## 3. Antibodies Targeted to Inducible Cancer Antigens

**Figure 1:**
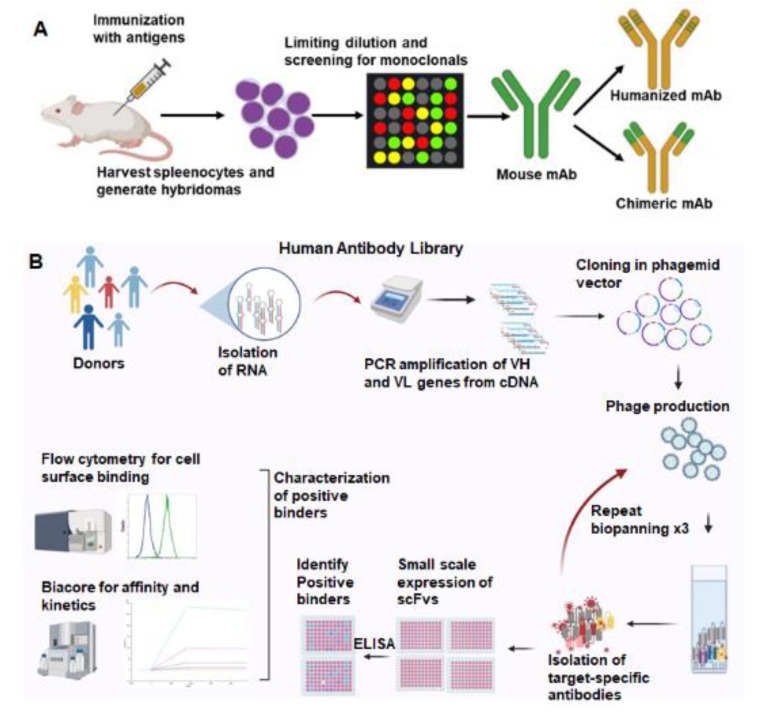
Two commonly used strategies for therapeutic antibody generation. (**A**). Mouse monoclonal antibodies are generated by immunizing mice with the target antigen. Splenocytes are harvested from immunized mice and fused with myeloma cells to create hybridomas. Selection and screening leads to identification of monospecific hybridoma clones that produce mouse monoclonal antibodies. Fv region of the human antibody backbone can be swapped with the murine Fv, generating a chimeric antibody. (**B**). Phage-displayed human antibody library screening can yield human antibodies against the target. Libraries are created by isolating RNA from peripheral blood and spleens of healthy donors. VH and VL genes are amplified from the cDNA and cloned into phagemid vectors. Phage-expressing antibodies on their surface were produced. Multiple rounds of phage-antibody selection are performed to isolate target-specific antibodies. Positive binders are screened by ELISA, flow cytometry, and affinity to select the lead antibodies. Created with BioRender.com.

We use two strategies to select antibodies to induce cancer antigens (Figure 1). Mouse monoclonal antibodies are produced by hybridoma technology, and a lead antibody is selected for humanization (Figure 1A). Secondly, bacteriophage-displayed human antibody libraries are used for biopanning (Figure 1B). Mouse monoclonal antibodies are selected from hybridomas isolated from mice inoculated with the inducible proteins. These antibodies are prioritized based on cancer-specific binding and high affinity for the cancer antigen. Murine antibodies have limited efficacy in humans due to a human anti-mouse antibody (HAMA) response that accelerates antibody clearance from the body. Antibody engineering techniques allow the humanization of murine antibodies to maintain target specificity and minimize the HAMA response. The antigen-binding region (CDR region) of lead murine antibodies is grafted into human IgG1. Human antibodies are also being generated using hybridoma technology in transgenic mice models, HuMabMouse and XenoMouse, where the mouse immunoglobulin (Ig) gene loci are replaced with human loci within the transgenic mouse genome [25].

An alternative to hybridoma technology is a human antibody phage display. Our antibody libraries were created from lymphocyte DNA donated from patients undergoing splenectomy or peripheral blood lymphocytes. The DNA-encoding antibodies are cloned into a phagemid vector, and the antibody variable region is expressed on bacteriophage fiber proteins. The resulting antibody library has more than 10^8^ distinct single-chain fragments of the variable (scFv) region of human antibodies. Biopanning is performed to select antibodies binding to the inducible antigens listed above. Cell-based biopanning is often utilized to maintain membrane proteins in their native conformation. scFv antibodies are then prioritized after they demonstrate cancer-specific binding and high affinity for the inducible antigen. Antibodies are then improved by affinity maturation and a reduction in immunogenicity. Several phage-display-derived antibodies have been approved in clinical trials [26]. Each of these approaches to antibody development results in functional human antibodies.

## 4. Mechanisms of Action of Cancer Therapeutic Antibodies

**Figure 2:**
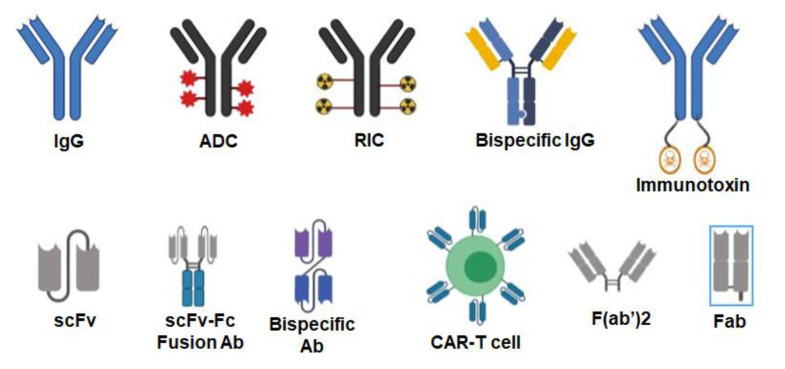
Various formats of antibodies. Antibodies can be used as naked IgG or conjugated to various agents such as drugs (antibody–drug conjugates or ADC), radioisotopes (radioimmunoconjugates or RIC) and toxins (immunotoxins). IgGs can be engineered to target two different antigens (bispecific IgG). Single-chain fragment variables (scFv) can be fused to the IgG Fc domain to generate bivalent scFv–Fc fusion antibodies. scFvs targeting two different antigens can be fused to each other with a linker making them a bispecific antibody. T-cells can be engineered into chimeric antigenic receptor (CAR)-T cells where an scFv is expressed on their surface to target the desired antigen. Smaller antibody formats such as F(ab’)2 and Fab do not contain the Fc region of the IgG. Created with BioRender.com.

A single therapeutic antibody can be developed into several different therapeutic agents that include antibody–drug conjugates (ADC), radioimmunoconjugates (RIC), bispecific antibodies (BsAbs), fusion antibodies, CAR T-cells, immunotoxins, and other agents (Figure 2).

**Figure 3:**
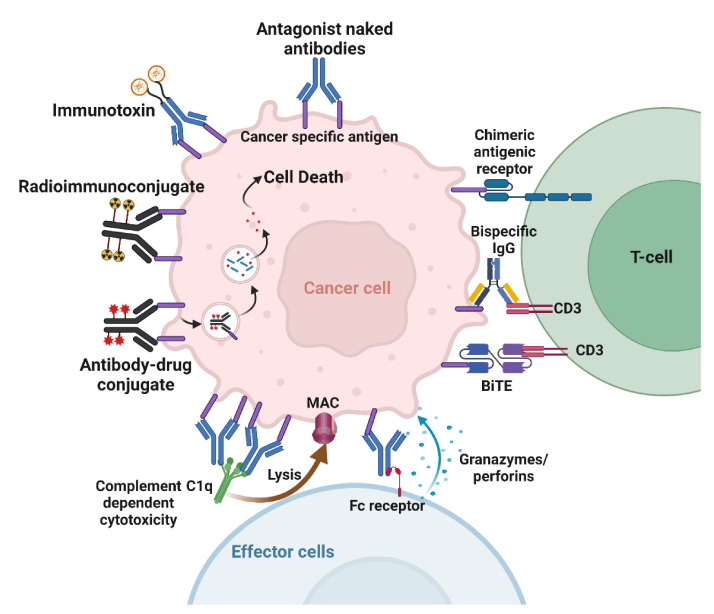
Mechanisms of action of therapeutic antibodies. Naked antibodies can cause cell death by acting as antagonists or by activating the immune effector cells. Immunoconjugates deliver payloads to the tumor. Bispecific antibodies and CAR crosslink tumor cells to T-cells. Created with BioRender.com.

Naked antibodies targeting cancer cells can cause cell death through various mechanisms. The best-known direct mechanism of cancer cell killing is blocking growth factor receptor signaling by antibodies. Antibody binding to receptors may block ligand binding, leading to downstream signaling inhibition (Figure 3). Cetuximab, an anti-epidermal growth factor receptor (EGFR) mAb, induces apoptosis in tumor cells by blocking ligand binding and receptor dimerization [27]. Antibodies targeting human epidermal growth factor receptor 2 (Her2) achieve signaling perturbation by inhibiting hetero-dimerization and internalization. The mechanism of action of antibodies targeting growth factor receptors is different from that of small-molecule tyrosine kinase inhibitors (TKIs). In contrast to TKIs that specifically target activating mutations in the kinase domain, and are therefore effective in a subset of patients, antibodies provide clinical benefit to all patients that over-express the targeted antigen irrespective of its mutation status [28,29,30]. Another advantage of antibodies over TKIs is their ability to activate immune effector cells as an additional mechanism to cell killing [28,29,30]. Antibodies can also inhibit tumor angiogenesis. Indirect mechanisms of action of mAbs need the engagement of the host immune system. These include complement-dependent cytotoxicity (CDC), antibody-dependent cellular phagocytosis (ADCP), and antibody-dependent cellular cytotoxicity (ADCC) (Figure 3). Most targeted mAbs can activate the complement system. Antibodies such as rituximab and ofatumumab activate the complement cascade, enhancing their antitumor efficacy. ADCP occurs when FcγRI, expressed on immune cells such as macrophages, binds to IgG1 or IgG3 mAbs that have opsonized a tumor cell. ADCP plays a vital role in destroying circulating tumor cells following mAb therapy [31]. Antibodies act as bridges by binding to antigens on the target cell surface via their Fab portions and linking the effector cells via their Fc portions.

Similarly, ADCC is an immune mechanism where target cells become opsonized by antibodies, which then recruit effector cells to induce target cell death by non-phagocytic mechanisms [32]. NK cells are the primary effector type that mediates ADCC; however, other myeloid types such as monocytes, macrophages, neutrophils, eosinophils, and dendritic cells are also capable. Effector cells induce target cell death via cytotoxic granule release, Fas signaling, and the initiation of reactive oxygen species [32].

Immunotoxins combine the specificity of mAbs with powerful cellular poisons derived from plants or microorganisms (Figure 3). An immunotoxin against mesothelin was tested in combination with radiation. The results show that the immunotoxin combined with either low or high doses of tumor-focused radiation led to superior antitumor activity [33]. Possible mechanisms that caused this effect were suggested to be enhanced tumor delivery of the immunotoxin or the upregulation of the surface expression of mesothelin following radiation [33].

Using radioimmunoconjugates is another approach that delivers cytotoxic agents to inducible antigens in cancer (Figure 3). Antibodies can be labeled with chelating molecules, to which radionuclides can be added. Targeting RICs to radiation-inducible cancer antigens is counterintuitive if merely conjugated to a β-emitter, as it would simply add a low LET internal emitter on top of low LET external irradiation. By way of contrast, conjugating high LET α-emitters or Auger-emitters to antibodies can specifically target very cytotoxic agents to cancer. For example, actinium-225 is an alpha emitter that releases four alpha particles per decay with a 10-day half-life [34,35]. This half-life is well-suited for antibodies with long circulation times. We conjugated the chelator DTPA to anti-TIP1 antibodies and radiolabeled them for imaging and dosimetry [36]. Cancer-selective binding of these RICs was accomplished. We also conjugated the antibodies to actinium-225 and measured the biodistribution in mouse models of lung cancer.

Antibody–boron conjugates are another example of antibody-mediated drug delivery to cancer. One new consideration is boron–proton capture therapy (BPCT), which is in early-stage drug development [37]. BPCT involves the interaction of an accelerated proton with ^11^B, resulting in the production of an excited ^12^C atom. This excited carbon-12 splits into an alpha particle and an excited ^8^Be atom, which subsequently degrades into two additional alpha particles. Thus, BPCT yields three alpha particles for each boron–proton capture. By way of contrast, boron–neutron capture therapy (BNCT) requires a ^10^B atom to capture a neutron resulting in the production of an alpha particle and ^7^Li atom. Antibodies to cancer-specific antigens can improve the bioavailability of boron conjugates and reduce normal tissue toxicities. Currently, we are conjugating larger quantities of boron to antibodies using dendrimers and nanoparticles. This approach will increase the amount of boron within cancer during boron neutron/proton capture therapy [38].

Antibodies can be conjugated to the surface of nanoparticles, similarly to how drug-encapsulated liposomes can deliver drugs such as an antibody–drug conjugate. In this case, the drug does not need to be directly conjugated to the antibody, and such particles may enhance drug delivery. Antibody-conjugated nanoparticles are reviewed in detail [39,40,41].

Bispecific antibodies (BsAbs) have gained more attention as a novel strategy for antitumor immunity, since they can simultaneously bind two different antigens (Figure 3). There are more than a hundred different BsAb formats described in the literature, but they can be broadly classified into two types: IgG-like format and Fc-free format [42]. An emerging application of BsAbs is radionuclide delivery. Instead of direct coupling to an antibody, a bsAb with an affinity for the tumor antigen and the radionuclide can be incubated with the payload before injection. Pretargeted delivery could also be achieved by first injecting the bsAb, and then injecting the payload. Pretargeting techniques to deliver radionuclides to a tumor circumvent the prolonged exposure of healthy tissue to the radionuclide, thus mitigating toxicity and adverse effects. This approach has been used to successfully deliver yttrium-90 [43,44].

## 5. Radiosensitizing Therapeutic Antibodies

Radiation-sensitizing antibodies can improve therapeutic outcomes while minimizing treatment-related adverse events such as lymphocyte depletion. Therapeutic antibodies can improve cancer control while minimizing lymphopenia, which is associated with the use of concomitant myelosuppressive chemotherapy [45,46]. The classic example of therapeutic antibody enhancement of radiotherapy efficacy is the use of cetuximab in the treatment of head and neck cancer [2]. Cetuximab binds specifically to the EGF receptor in squamous cell carcinomas and improves the efficacy of radiotherapy. Preclinical studies of antibody–drug conjugates have also been shown to improve the cytotoxic effects of radiotherapy [3,4,5]. Thus, therapeutic antibodies can serve as radiosensitizing drugs.

**Figure 4:**
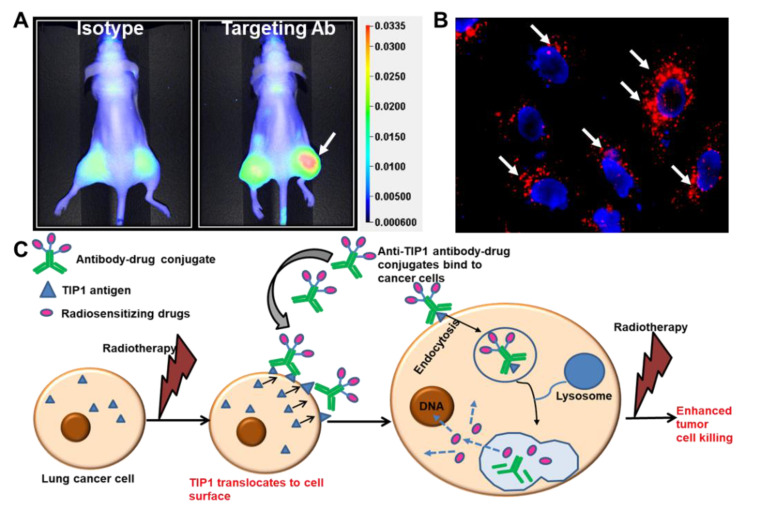
Radiation-sensitizing antibody–drug conjugates. (**A**) Near-infrared (NIR) imaging showing specific binding of the antibody targeting a radiation-inducible antigen to irradiated tumors (white arrow). Nude mice were injected subcutaneously with A549 cells. The right hind limb tumors were irradiated with three doses of 3 Gy. Isotype control or targeting antibody was injected via tail vein, and NIR imaging was performed to evaluate the biodistribution of the antibody (Adapted from [3]). (**B**) Endocytosis of ADC in cancer cells. The cancer-specific antibody was labeled with pH-sensitive dye pHrodo and incubated with cancer cells. Punctate red fluorescence indicates accumulation of the antibody in acidic compartments of the cells (white arrows). Nuclei are stained blue. (**C**) Schematic representation of the strategy to enhance the therapeutic efficacy of radiation and reduce detrimental side effects. Radiation enhances surface expression of radiation inducible antigens such as TIP1 in lung cancer and not normal cells, leading to specific binding of ADCs to cancer. ADCs deliver radiosensitizers to cancer. Radiotherapy then leads to enhanced cancer cell killing without effecting normal cells.

Similar to cetuximab, antibodies to the radiation-inducible proteins GRP78 and TIP1 enhance the cytotoxic effects of radiotherapy and improve survival in mouse models of cancer [15]. Cancer cell surface expression of these stress-regulated proteins increases their accessibility to antibody binding during radiotherapy. We studied cancer-specific binding using three approaches. Firstly, antibodies labeled with imaging agents allow for whole-animal imaging using mouse models of cancer. This approach is useful when the mouse target protein is homologous to that of the human protein, as is the case for GRP78 and TIP1. Figure 4A shows whole-animal imaging following labeled antibody administration to mice bearing irradiated cancers (Figure 4A) [3]. Secondly, we studied antibody binding to irradiated cells from normal human tissues. Finally, we measured antibody binding to tissues from all organs (tissue cross-reactivity). Antibodies to the functional domains of both GRP78 and TIP1 show cancer-specific binding in each of these studies.

GRP78 is a radiation-inducible cancer cell surface protein discovered using the phage-displayed peptide library injected into the circulation [20]. Antibodies to the functional domain of GRP78 induce cancer-specific cytotoxicity in mouse models of human cancers [13,47]. We studied the efficacy of anti-GRP78 antibodies in combination with radiotherapy. Antibodies to the functional domain of GRP78 disrupted its interaction with binding partners, reducing cancer cell viability and radiosensitization. This approach improved survival in mouse models of human glioblastoma and lung cancer [15]. Humanized anti-GRP78 antibodies targeting the functional domain of GRP78 are in preclinical drug development.

TIP1 is a radiation-inducible antigen discovered by the phage display peptide library. TIP1 expression levels correlate with worsened outcomes in human cancers [48]. TIP1 is a scaffold protein that anchors signaling proteins to the cell membrane. TIP1 is translocated to the cancer cell surface during the stress response to radiation, where it is accessible to antibody binding [36]. We have found that antibodies to this inducible antigen enhance the efficacy of radiotherapy. The functional domain (PDZ domain) of TIP1 binds proteins that play an essential role in cancer cell viability and migration [48,49]. Antibodies to the PDZ domain disrupt TIP1 interaction with binding partners, reducing cancer cell viability and radiosensitization. Mouse models with human cancers showed improved survival when anti-TIP1 antibodies were administered during radiotherapy.

## 6. Antibody–Drug Conjugates

Antibody–drug conjugates (ADCs) deliver cytotoxic drugs that are specific to cancer. ADCs are emerging as a rapidly expanding class of therapeutic agents used for cancer treatment. In fact, twelve different ADCs are FDA-approved to treat multiple cancer subtypes, and various ADCs are in the pipeline for clinical development [50,51,52,53,54]. There are three components to ADCs: antibody, drug, and linker. Linkers are used to conjugate the drug to the antibody. Following endocytosis, the drug dissociates from the antibody and initiates cytotoxicity. Antibodies can be conjugated to radiation-sensitizing drugs. This approach has been shown to enhance the efficacy of radiotherapy [3,4,5,55,56]. In those studies, monomethyl auristatin E (MMAE) or emtansine (DM1) were conjugated to anti-EGFR and Herceptin to achieve radiation sensitization in pre-clinical cancer models [4,56]. A pre-clinical assessment of ADC with radiotherapy was conducted in mouse models of human pancreatic cancer. Anti-HER3-MMAE enhanced radiotherapy and improved tumor control [55]. The efficacy of anti-EGFR antibody conjugated to two different radiation sensitizing drugs, MMAE and DM1, achieved radiosensitization of preclinical models of lung, head and neck, and esophageal cancers. Mouse models of human cancer showed substantial tumor control and improved survival when ADC was combined with radiotherapy. This group also targeted HER2 on human cancers using antibody conjugates of MMAE. This approach showed enhanced efficacy and improved survival in mouse models of human cancer [5].

The use of ADCs as radiosensitizers has been validated by several studies. Most notable is the phase I clinical trial evaluating the safety of ADC with radiotherapy in patients with newly diagnosed glioblastoma [57]. Anti-EGFR antibody conjugated to MMAF (ABT414) was administered to glioblastoma patients receiving standard-of-care radiotherapy and TMZ. This agent was well tolerated in dose escalation up to 3.3 mg/kg. In our recent publication, we found that the anti-TIP1 antibodies undergo endocytosis following binding on the surface of cancer cells [3] (Figure 4B). We conjugated the chemotherapeutic agent monomethyl auristatin E (MMAE) to anti-TIP1 antibodies and measured its enhancement of tumor control in preclinical models of irradiated cancer. The ADC increased MMAE delivery to cancer, enhanced radiation-induced cytotoxicity, and improved tumor control. Figure 4C shows a schematic representation of our proposed approach to guide the delivery of radiation-sensitizers by targeting radiation-inducible antigens. In pancreatic ductal adenocarcinoma (PDAC), an MMAE conjugated, anti-HER3-ADC increased response to radiation through the inhibition of cell survival and the induction of DNA break formation and apoptosis [55]. Another auristatin derivative, monomethyl auristatin F (MMAF), was conjugated to an anti-HER2 antibody, and this conjugate also improved tumor control combined with focal ionizing radiation [5]. MMAF had the advantage of decreased bystander and off-target effects compared with MMAE [5]. Thus, it is feasible to deliver radiosensitizing drugs specifically to irradiated cancer, thereby improving bioavailability and reducing systemic toxicities.

## 7. Planned Clinical Trials

Radiation-inducible antigens for cancer drug delivery have not been explored in clinical trials. However, the efficacy and safety results of an ADC were evaluated in combination with radiation and temozolomide in newly diagnosed glioblastoma [57]. Anti-EGFR antibody conjugated to MMAF (ABT414) was administered to glioblastoma patients receiving standard-of-care radiotherapy and TMZ. This agent was well tolerated in dose escalation up to 3.3 mg/kg. The first goal of our planned clinical trials is to learn the safety profile and the pharmacokinetics of the therapeutic antibodies. Anti-TIP1 IgG will be administered to three cohorts, including patients with metastatic cancer, those receiving palliative irradiation, and a third cohort treated with checkpoint blockade antibodies. ^89^Zr-antibody imaging studies are well-established for evaluating antibody safety and pharmacokinetics [58]. We will image the biodistribution of antibodies labeled with ^89^Zr using PET imaging. The long-term goals include studies of efficacy and reduction in myelosuppression in cancer patients receiving radiotherapy with antibody therapy. Radiation-inducible antigens represent an opportunity for the radiation oncology community to participate in cancer drug development. We welcome comments on planned clinical trials.

In summary, the radiation induction of cancer antigens markedly increases the number of antigens well suited for therapeutic antibody development. These antibodies can be used to deliver radiosensitizing drugs. This new clinical paradigm of using radiation to guide drug delivery will enter clinical trials in radiation oncology patients with poor-prognosis cancers.
